# Oncogenic driver FGFR3-TACC3 requires five coiled-coil heptads for activation and disulfide bond formation for stability

**DOI:** 10.18632/oncotarget.28359

**Published:** 2023-02-11

**Authors:** Clark G. Wang, Malalage N. Peiris, April N. Meyer, Katelyn N. Nelson, Daniel J. Donoghue

**Affiliations:** ^1^Department of Chemistry and Biochemistry, University of California San Diego, La Jolla, CA 92093, USA; ^2^Biomedical Sciences Graduate Program, University of California San Diego, La Jolla, CA 92093, USA; ^3^Moores UCSD Cancer Center, University of California San Diego, La Jolla, CA 92093, USA

**Keywords:** oncogenic fusion protein, chromosomal translocation, glioblastoma, FGFR3-TACC3, coiled-coil

## Abstract

FGFR3-TACC3 represents an oncogenic fusion protein frequently identified in glioblastoma, lung cancer, bladder cancer, oral cancer, head and neck squamous cell carcinoma, gallbladder cancer, and cervical cancer. Various exon breakpoints of FGFR3-TACC3 have been identified in cancers; these were analyzed to determine the minimum contribution of TACC3 for activation of the FGFR3-TACC3 fusion protein. While TACC3 exons 11 and 12 are dispensable for activity, our results show that FGFR3-TACC3 requires exons 13-16 for biological activity. A detailed analysis of exon 13, which consists of 8 heptads forming a coiled coil, further defined the minimal region for biological activity as consisting of 5 heptads from exon 13, in addition to exons 14-16. These conclusions were supported by transformation assays of biological activity, examination of MAPK pathway activation, analysis of disulfide-bonded FGFR3-TACC3, and by examination of the Endoglycosidase H-resistant portion of FGFR3-TACC3. These results demonstrate that clinically identified FGFR3-TACC3 fusion proteins differ in their biological activity, depending upon the specific breakpoint. This study further suggests the TACC3 dimerization domain of FGFR3-TACC3 as a novel target in treating FGFR translocation driven cancers.

## INTRODUCTION

Fibroblast growth factor receptors (FGFRs) are members of the receptor tyrosine kinase (RTK) family that regulates developmental processes including cellular proliferation and cell differentiation. Normally, fibroblast growth factors (FGFs) and heparin sulfate proteoglycans bind to the extracellular domain of FGFRs, causing dimerization and subsequent trans-autophosphorylation of tyrosine residues within the intracellular kinase domain of FGFRs, leading to activation of downstream signaling pathways. However, mutations of FGFRs often lead to various developmental disorders and cancers [1]. FGFR chromosomal translocations have been identified in both solid and hematological malignancies. These translocations lead to active oncogenic fusion proteins that are believed to dimerize via the dimerization domain of the fusion partner rather than binding of FGF ligands. Ultimately, dimerization of these fusion proteins results in constitutive FGFR activation and stimulation of downstream cell signaling pathways such as JAK/STAT, Ras/MAPK, P13K/Akt, and PLCγ [2–4].

This work focuses on FGFR3-TACC3, a fusion between fibroblast growth factor receptor 3 (FGFR3) and transforming acidic coiled-coil containing protein 3 (TACC3). As the result of an intrachromosomal translocation on human chromosome 4p16, FGFR3-TACC3 was first discovered in glioblastoma with TACC3 fused to a slightly truncated FGFR3 with an intact kinase domain [5]. FGFR3-TACC3 is suggested to dimerize through the coiled-coil domain present in TACC3, resulting in constitutive activation of the FGFR3 kinase domain, and leading to the activation of the Ras/MAPK and other signaling pathways [3]. Others have shown that the loss of the 3′ untranslated region of FGFR3 allows the fusion to escape miR-99a regulation in glioblastoma; additionally, the TACC3 fusion partner induces mitotic defects by recruiting endogenous TACC3 away from the mitotic spindle, where TACC3 is canonically required for providing stabilization [6, 7]. Previously we have shown that FGFR3-TACC3 requires either entrance to the secretory pathway or plasma membrane localization for oncogenic activation [8].

The FGFR3-TACC3 fusion protein has been identified with differing exonic breakpoints within TACC3 in numerous cancers including glioblastoma, bladder cancer, lung adenocarcinoma, and head and neck squamous cell carcinoma [1]. Although tyrosine kinase inhibitors (TKIs) are often used to treat these dysregulated FGFR driven cancers, this therapeutic method often leads to the emergence of a resistant population of cancer cells exhibiting gatekeeper mutations [9]. Such mutations prevent proper binding of TKIs, giving way to a subset of cancer cells that are resistant to TKI treatment, ultimately leading to relapse in patients despite initial responsiveness to treatment [10]. This developed resistance to TKI therapies highlights the need for new approaches to treating cancers with dysregulated FGFRs.

The disruption of dimerization via the inhibition of a protein’s dimerization domain presents a novel strategy for treating oncogenic fusion proteins. The disruption of the oligomerization domain in BCR-ABL and BCR-FGFR1, fusion proteins identified as drivers of cancer in leukemia, has demonstrated a decrease or loss in cell transformation [11, 12]. Rationally designed coiled-coil mimetics, that competitively bind to the coiled-coil regions of the BCR fusion partner, resulted in a reduction in transformation ability of BCR-ABL expressing cells [13]. This highlights the significance of characterizing dimerization partners of fusion proteins, as they could become new targets for therapeutic development.

Disulfide bonds are usually found to provide structural stability for proteins that are exposed to the oxidizing environment outside of the cell. However, disulfide bond formation has the potential to regulate protein function within the cell. In some cases, these disulfide bonds instead respond to oxidative stress in a reversible manner, driven by interactions with reactive oxygen species (ROS), allowing them to function as redox switches. ROS has been found to regulate apoptosis and other cellular processes; for example, ROS can alter disulfide-mediated interactions between caspase-9 and Apaf-1 [14]. Intracellular oxidative stress has been shown to massively stimulate protein disulfide bond formation in the cytoplasm [15]. Furthermore, FGFR3-TACC3 has been shown to activate oxidative phosphorylation and mitochondrial biogenesis through a mechanism involving Pin4 phosphorylation, which leads to peroxisomal biogenesis and the production of intracellular ROS [16].

In this work, we characterize the signaling, transforming abilities, and post-translational modifications of FGFR3-TACC3 fusion proteins arising from different exonic breakpoints to determine the requirements for dimerization and constitutive activation of the fusion protein.

## RESULTS

### Expression of FGFR3-TACC3 fusion proteins of varying lengths

**Figure 1:**
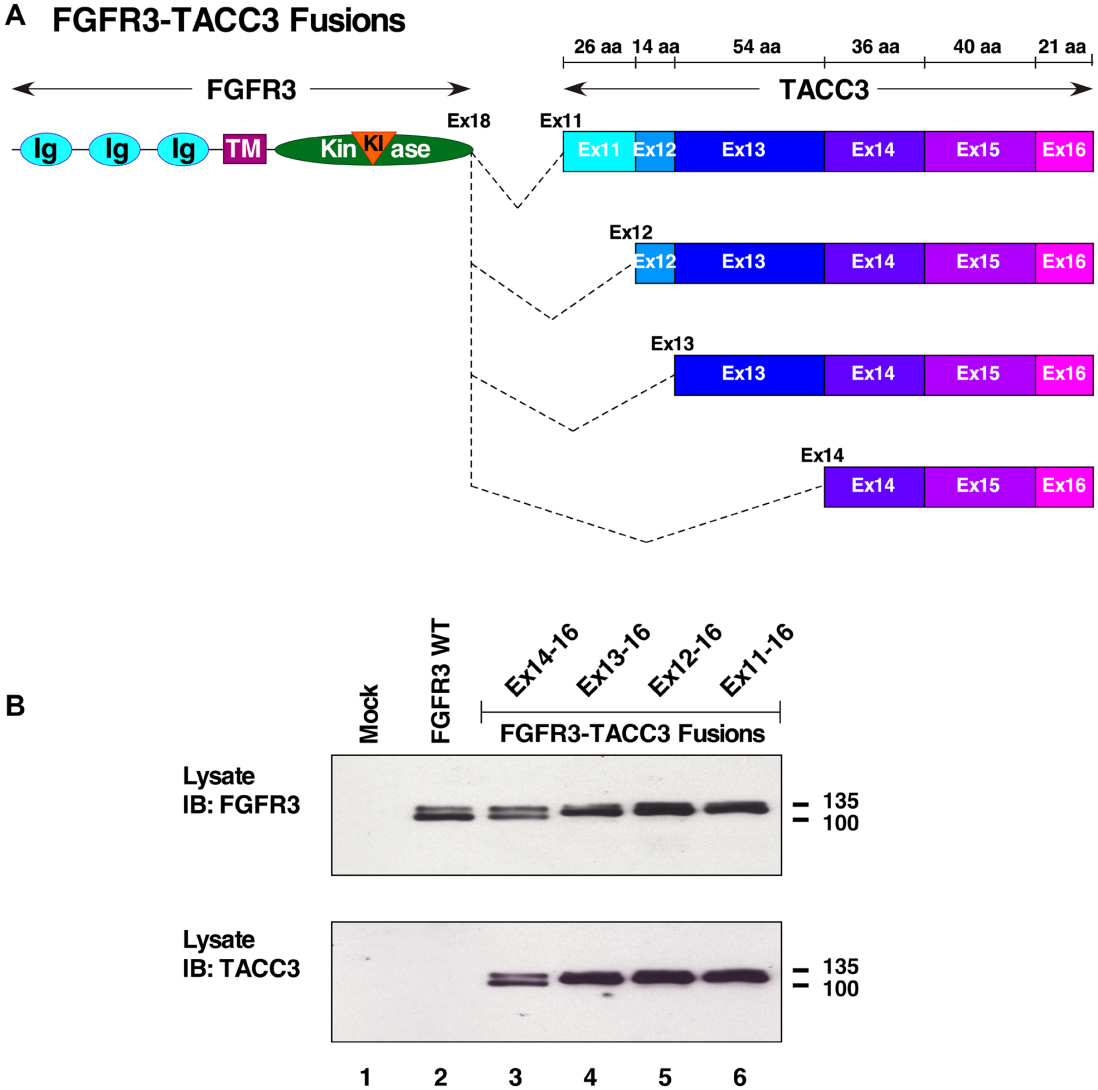
FGFR3-TACC3 fusion proteins. (**A**) Schematic of FGFR3-TACC3 breakpoints with 3′ gene fusion points of TACC3 at exons 11, 12, 13, and 14 fused to FGFR3 exon 18. N-terminal extracellular ligand binding domain of FGFR3 containing IG-like (Ig) domains, transmembrane (TM), kinase and kinase insert (KI) domains fused to TACC3 coiled-coil domain. (**B**) HEK293T cell lysates expressing FGFR3 or FGFR3-TACC3 fusions were immunoblotted with antisera to detect the N-terminus of FGFR3 (top panel). The membrane was stripped and re-probed with antisera to detect the C-terminus of TACC3 (bottom panel). Molecular weight markers are indicated in kDa.

The FGFR3-TACC3 fusion protein has been identified with various gene fusion points, resulting in differing lengths of the dimerization partner, TACC3. A collection of these breakpoints was selected for analysis (Figure 1A). These breakpoints included: FGFR3-TACC3 Ex11-16, in which exons 11-16 encode 191 amino acids; FGFR3-TACC3 Ex12-16, in which exons 12-16 encode 165 amino acids; FGFR3-TACC3 Ex13-16, in which exons 13-16 encode 151 amino acids; and FGFR3-TACC3 Ex14-16, in which exons 14-16 encode only the C-terminal 97 amino acids of TACC3. These constructs were validated by DNA sequencing and by expression in HEK293T cells, followed by immunoblotting of cell lysates with an antiserum recognizing the N-terminus of FGFR3 (Figure 1B, top), or with an antiserum recognizing the C-terminus of TACC3 (Figure 1B, bottom).

FGFR3-TACC3 proteins have been identified in many cancers including glioblastoma, bladder cancer, and head and neck squamous cell carcinoma [1, 17, 18]. FGFR3-TACC3 Ex11-16 is the most common fusion identified in patients, followed by FGFR3-TACC3 Ex8-16, while FGFR3-TACC3 Ex13-16 has been identified on three occasions [18, 19]. FGFR3-TACC3 Ex14-16, the shortest form to be observed clinically, has been reported once in a head and neck tumor [17]. Although FGFR3-TACC3 Ex12-16 has not been identified in patients, it was constructed as an intermediate breakpoint for experimental purposes. The algorithm Multicoil2 predicted TACC3 to contain multiple coiled-coil domains, starting in exon 10 of TACC3 onwards to the end of the protein in exon 16 [20].

### Oncogenic potential of FGFR3-TACC3 fusion proteins

**Figure 2:**
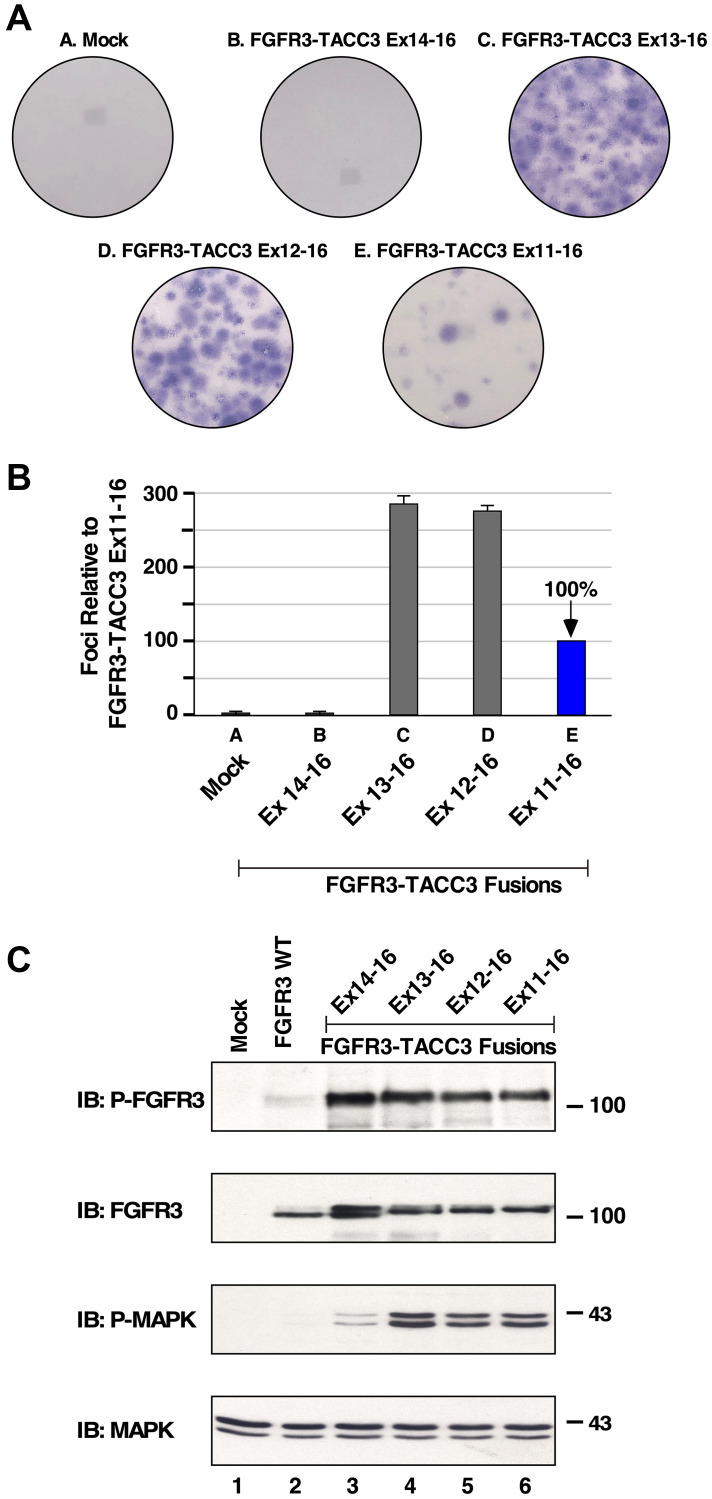
Transformation and downstream signaling of FGFR3-TACC3 fusion proteins. (**A**) Representative plates of NIH3T3 cells show transformation by FGFR3-TACC3 fusion proteins in a focus formation assay. (**B**) Numbers of foci were counted, normalized for transfection efficiency relative to the FGFR3-TACC3 Ex11-16 fusion [3], and presented as a percentage of transformation +/− SEM relative to FGFR3-TACC3 Ex11-16. (**C**) HEK293T cell lysates expressing FGFR3 or FGFR3-TACC3 derivatives were immunoblotted for Phospho-FGF receptor (P-FGFR) top panel, FGFR3 2nd panel, Phospho-p44/42 MAPK (P-MAPK) 3rd panel, and p44/42 Erk1/2 (MAPK) 4th panel. Molecular weight markers are indicated, kDa.

Previously, FGFR3-TACC3 with breakpoints at exon 8 and exon 11 were shown to exhibit oncogenicity by cellular transformation of NIH3T3 cells [7]. To analyze biological activity, NIH3T3 cells expressing the new FGFR3-TACC3 fusion proteins were assayed for focus forming ability. As shown in Figure 2A and quantitated in Figure 2B, three of these fusion proteins exhibit potent biological activity: FGFR3-TACC3 Ex13-16, Ex12-16, and Ex11-16; interestingly, FGFR3-TACC3 Ex14-16 was devoid of activity in this assay. These results suggest that FGFR3-TACC3 Ex14-16, despite being identified in a head and neck tumor [17], may not be biologically active and may require additional mutations for cellular transformation.

### Downstream signaling of FGFR3-TACC3 fusion proteins

It has been previously shown that the FGFR3-TACC3 Ex8-16 and FGFR3-TACC3 Ex11-16 activate the Ras/MAPK pathway, likely due to activation and phosphorylation of the FGFR3 tyrosine kinase domain [3]. However, the downstream cell signaling and mechanism of activation of additional FGFR3-TACC3 breakpoints have not been examined previously. Therefore, HEK293T cells expressing various FGFR3-TACC3 fusions were collected, lysed and analyzed for activation of downstream cell signaling by immunoblotting. Each FGFR3-TACC3 breakpoint showed strong receptor activation loop tyrosine phosphorylation. As expected, FGFR3 wild-type exhibited no activation as detected by phospho-specific FGFR antisera (Figure 2C, top panel).

To further examine the signaling network employed by the FGFR3-TACC3 fusions, cell lysates were immunoblotted with P-MAPK antiserum to detect activation of the Ras/MAPK pathways (Figure 2C, 3rd panel). Interestingly, each FGFR3-TACC3 fusion protein showed strong activation of MAPK signaling; the only exception was FGFR3-TACC3 Ex14-16 which exhibited a very weak level of phospho-MAPK (3rd panel, lane 3). This was in contrast to the phospho-FGFR3 signal detected for FGFR3-TACC3 Ex14-16 (1st panel, lane 3). These results suggest that FGFR3-TACC3 Ex14-16 may not be a driver of cancer, as this fusion protein is unable to activate a major downstream signaling pathway associated with cell growth and proliferation.

### Disulfide-bonded dimer in biologically active FGFR3-TACC3 fusions

**Figure 3:**
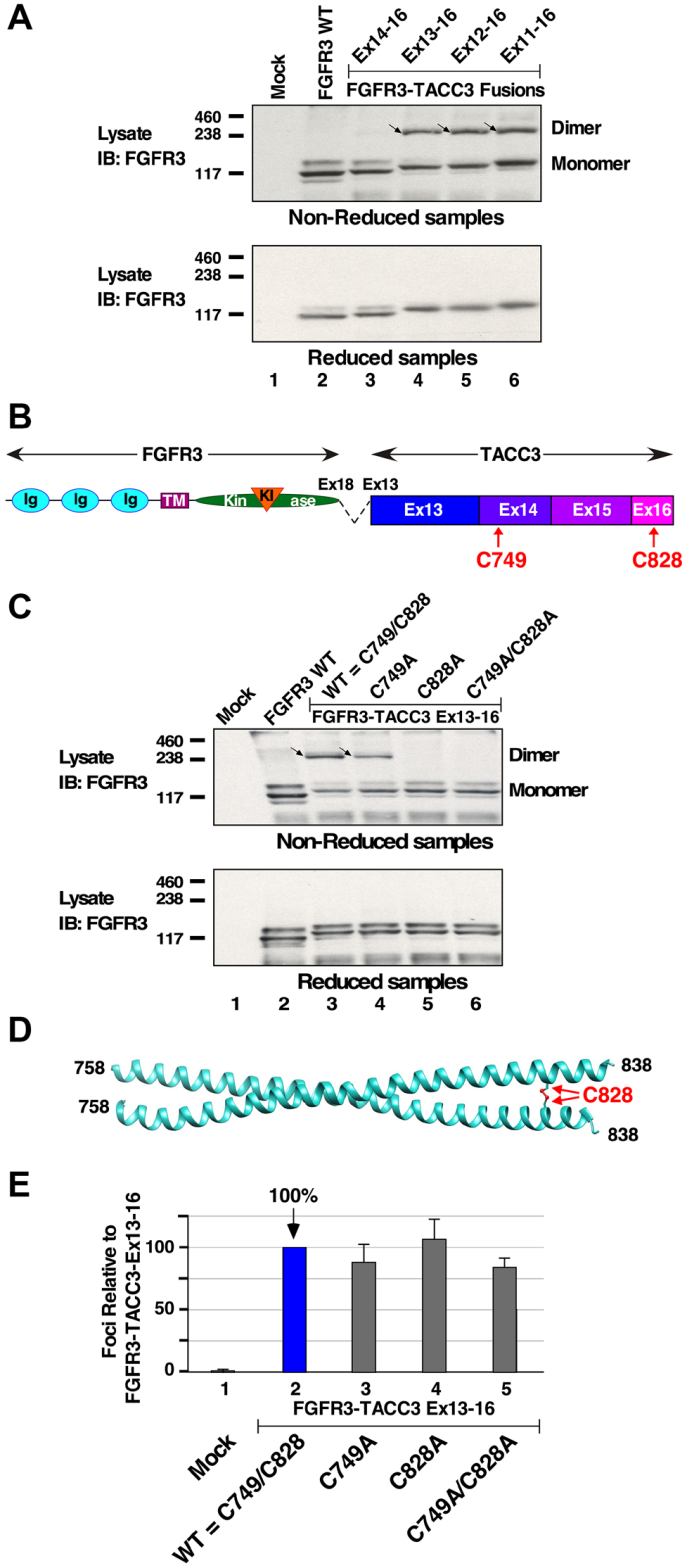
Disulfide-bonded dimer formation in FGFR3-TACC3 fusion proteins. (**A**) HEK293T cell lysates expressing FGFR3 or FGFR3-TACC3 derivatives were immunoblotted for FGFR3 in either non-reducing (top panel, dimers indicted by arrows) or reducing (lower panel) conditions. Molecular weight markers are indicated, kDa. (**B**) The locations of Cys residues C749 within Exon 14, and C828 within Exon 16, are shown. (**C**) HEK293T cell lysates expressing FGFR3 or derivatives of FGFR3-TACC3 Ex13-16 were immunoblotted for FGFR3 in either non-reducing (top panel, dimers indicted by arrows) or reducing (lower panel) conditions. Molecular weight markers are indicated, kDa. (**D**) 5LXN crystal structure of the C-terminus of TACC3 is shown with the disulfide bond at C828 highlighted [21]. (**E**) Transformation of NIH3T3 cells is presented for mutants at C749 and C828 of FGFR3-TACC3 Ex13-16. Numbers of foci were counted, normalized for transfection efficiency, and calculated +/− SEM as a percentage of transformation relative to FGFR3-TACC3 Ex13-16.

Variations in possible post-translational modifications were investigated due to differences detected in biological activity and downstream signaling activation between FGFR3-TACC3 Ex14-16 and other FGFR3-TACC3 fusion proteins. HEK293T cells expressing FGFR3-TACC3 fusion proteins were analyzed for possible disulfide-bonded proteins. When analyzed under nonreducing conditions, FGFR3 WT protein did not exhibit disulfide-bonded dimeric protein, as expected (Figure 3A, top panel, lane 2). Interestingly, non-reduced HEK293T lysates yielded disulfide-bonded dimers for each breakpoint (top panel, lanes 4–6). The exception was provided by FGFR3-TACC3 Ex14-16 (top panel, lane 3), which exhibited negligible disulfide-bonded dimer. When cell lysates were analyzed using conditions to reduce disulfide bonds, only monomers were observed as expected for each protein (Figure 3A, lower panel). This shows the discovery of one or more disulfide bonds that create covalently dimerized FGFR3-TACC3 fusion proteins.

Cysteine residues C749 and C828 were identified in the region spanning TACC3 Ex13-16. Importantly, within the entirety of Exons 13-16, C749 and C828 are the only cysteine residues present and, therefore, the only possible amino acids involved in disulfide bond formation (Figure 3B). To determine if these residues are important for the observed disulfide bond formation of FGFR3-TACC3 fusions, single mutations C749A or C828A, or a double mutation C749A/C828A, were introduced into FGFR3-TACC3 Ex13-16 and analyzed under non-reducing conditions by immunoblotting (Figure 3C, top panel). The FGFR3-TACC3 Ex13-16 containing the C749A mutation exhibited a decreased disulfide-bonded dimer population, while both the C828A single mutant as well as the C749A/C828A double mutant completely lost their disulfide-bonded dimers. These results indicate that C828 is absolutely required for disulfide bond formation of the FGFR3-TACC3 Ex13-16 fusion protein, but that C749 also contributes to a lesser extent. These results are consistent with a model in which C749 and C828 participate in disulfide bonds with their homologous residues of the dimeric partner. A partial crystal structure of TACC3 is available and includes the region spanning C828, shown as disulfide-bonded in Figure 3D; however, the region of FGFR3-TACC3 accessible in the crystal structure 5LXN does not include C749 [21].

To determine whether residues C749 and C828 are required for biological activity, NIH3T3 cells expressing derivatives of FGFR3-TACC3 Ex13-16 containing either the single mutations C749A or C828A, or the double mutation C749A/C828A, were analyzed in a focus formation assay. No significant change in transforming ability was observed (Figure 3E) compared to the parental FGFR3-TACC3 Ex13-16. Somewhat to our surprise, these results show that disulfide bond formation involving either C749 or C828 is not essential for biological activity of FGFR3-TACC3.

Due to the presence of disulfide bonds in FGFR-TACC3, which in other proteins may act as sensors for ROS [14–16, 22], we wished to determine if their formation is sensitive to ROS conditions in the cell. Therefore, HEK293T cells expressing either FGFR3-TACC3 Ex13-16, with or without the double mutation C749A/C828A to prevent disulfide bond formation, were treated with increasing concentrations of H_2_O_2_ or Diamide to induce intracellular ROS [15]. Protein lysates were then examined by immunoblotting for total FGFR3 and phospho-FGFR3 to determine whether intracellular ROS resulted in increased dimer formation or receptor activation. Despite the known role of FGFR3-TACC3 in stimulating the production of intracellular ROS [16], we were unable to demonstrate a significant increase in dimerization and/or activation in response to the induction of ROS (data not shown).

### Five coiled-coil heptad repeats are required for activation of FGFR3-TACC3

FGFR3-TACC3 Ex14-16 is not biologically active and unable to activate downstream Ras/MAPK signaling. However, with the addition of a single exon, FGFR3-TACC3 Ex13-16 retains signaling and transforming ability. FGFR3-TACC3 Ex13-16 exhibits a larger coiled-coil segment from the TACC3 protein, in comparison to FGFR3-TACC3 Ex14-16. Coiled-coils are structural motifs that often exhibit heptad repeats, or patterns of seven residues in the form of (HPPHPPP)_n_, with predominantly hydrophobic residues in H positions and predominantly polar residues in P positions. Multicoil2, an algorithm that identifies coiled-coils, was utilized to investigate the differences between biologically active FGFR3-TACC3 Ex13-16 and inactive FGFR3-TACC3 Ex14-16 [20].

**Figure 4:**
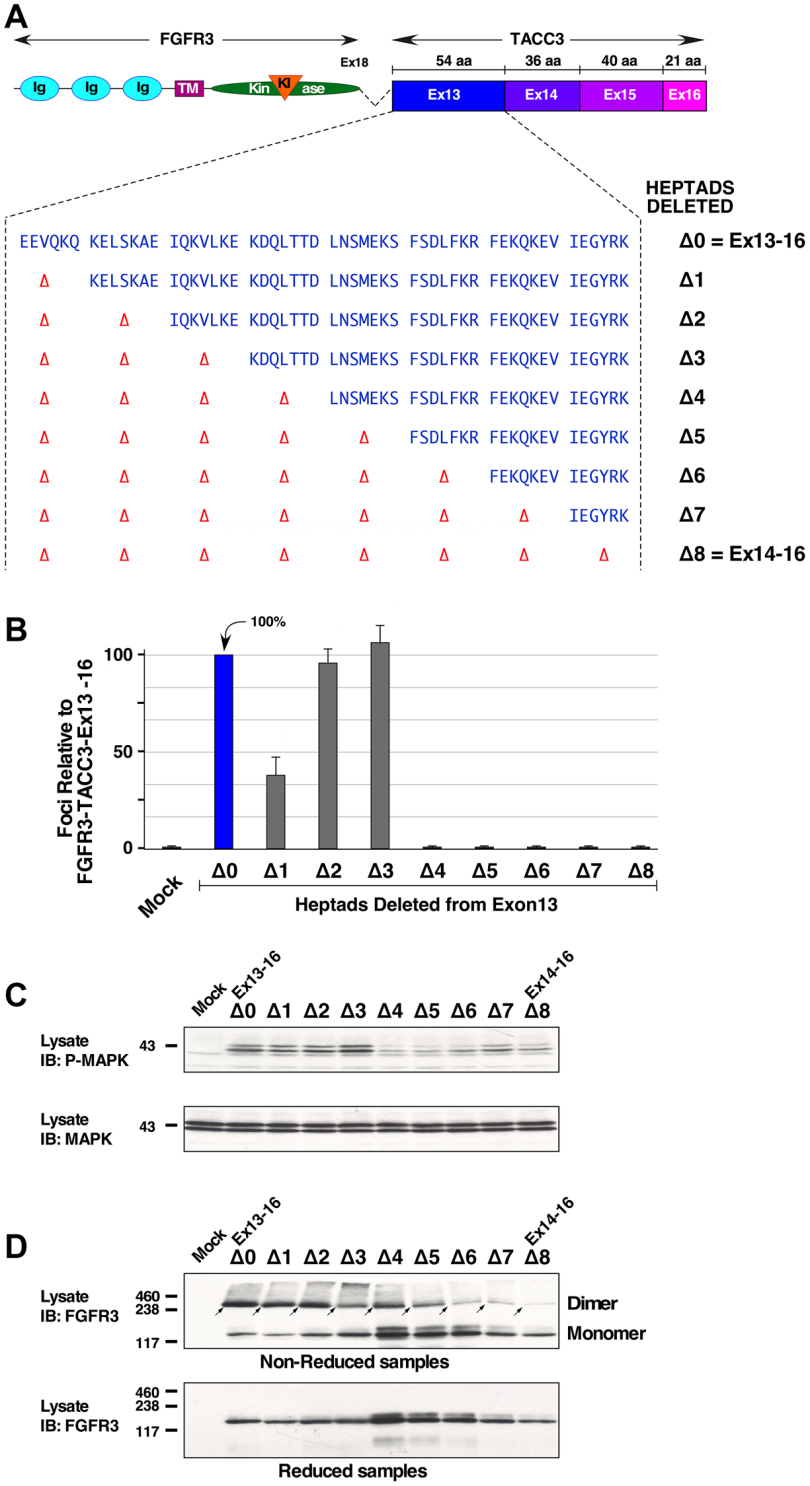
Heptad repeats required for activation and dimerization of FGFR3-TACC3 Ex13-16. (**A**) Schematic of FGFR3-TACC3 Ex13-16 with heptad amino acids shown. (**B**) Transformation of NIH3T3 cells by derivatives of FGFR3-TACC3 Ex13-16. Numbers of foci were counted, normalized for transfection efficiency, and calculated +/− SEM as a percentage of transformation relative to FGFR3-TACC3 Ex13-16. (**C**) HEK293T cell lysates expressing derivatives of FGFR3-TACC3 Ex13-16 were immunoblotted for Phospho-p44/42 MAPK (P-MAPK) 1st panel, and p44/42 Erk1/2 (MAPK) 2nd panel. Molecular weight markers are indicated, kDa. (**D**) HEK293T cell lysates expressing derivatives of FGFR3-TACC3 Ex13-16 were immunoblotted for FGFR3 in either non-reducing (top panel, dimers indicted by arrows) or reducing (lower panel) conditions. Molecular weight markers are indicated, kDa.

Coiled coils comprise a helical structure where each amino acid residue corresponds to a right handed rotation of ~100°C, such that a complete heptad deletion should remove two complete turns of the helix and return the remaining protein to its original rotational position [23]. Eight heptad repeats were identified in TACC3 exon 13, of which the first (EEVQKQ) contains only six residues (Figure 4A). To determine the number of heptads that are required for biological activity, NIH3T3 cells expressing FGFR3-TACC3 Ex13-16 with incremental deletions of heptads were analyzed in focus formation assays (Figure 4B). Cells expressing FGFR3-TACC3 Ex13-16 with a one heptad deletion (Δ1) partially reduced transformation (Figure 4B). The removal of the next two heptads (Δ2 and Δ3) had little or no effect on transformation. However, cells expressing FGFR3-TACC3 Ex13-16 with a deletion of 4 (Δ4) or more heptads led to complete abrogation of transformation. These results show that the remaining five heptads in the coiled-coil region of TACC3, present in the Δ3 mutant, are necessary for the activation of the FGFR3-TACC3 fusion protein. We believe the partial reduction in cellular transformation by deletion of the first heptad, Δ1, may be due to the fact that this “heptad” is in fact only six residues; thus its deletion would not completely restore the rotational alignment in a dimeric structure [23].

The complete set of heptad deletions was also examined for MAPK activation in HEK293T cells (Figure 4C), which showed a strong correlation with the NIH3T3 transformation assay. The Δ0, Δ1, Δ2, and Δ3 mutants showing elevated phospho-MAPK in comparison with the non-transforming mutants Δ4 through Δ8.

Cells expressing FGFR3-TACC3 Ex13-16 and its subsequent heptad mutants were also analyzed for disulfide-bonded dimers (Figure 4D). The incremental deletion of heptads led to a significant loss of the disulfide-bonded dimer population starting from the deletion of six heptads (Δ6) and onwards. A general trend is observed, i.e. progressive heptads deletion show reduced dimer formation (Figure 4D, top) and also show reduced biological activity (Figure 4B). However, the correlation is not perfect as shown by the Δ4 and Δ5 heptad deletions which exhibit dimer formation but are biologically inactive, for which we have no clear explanation. We can conclude, however, that progressive heptad deletion in exon 13 generally correlates with loss of disulfide-bonded dimers and with lack of transformation.

### Analysis of FGFR3-TACC3 proteoglycans

**Figure 5:**
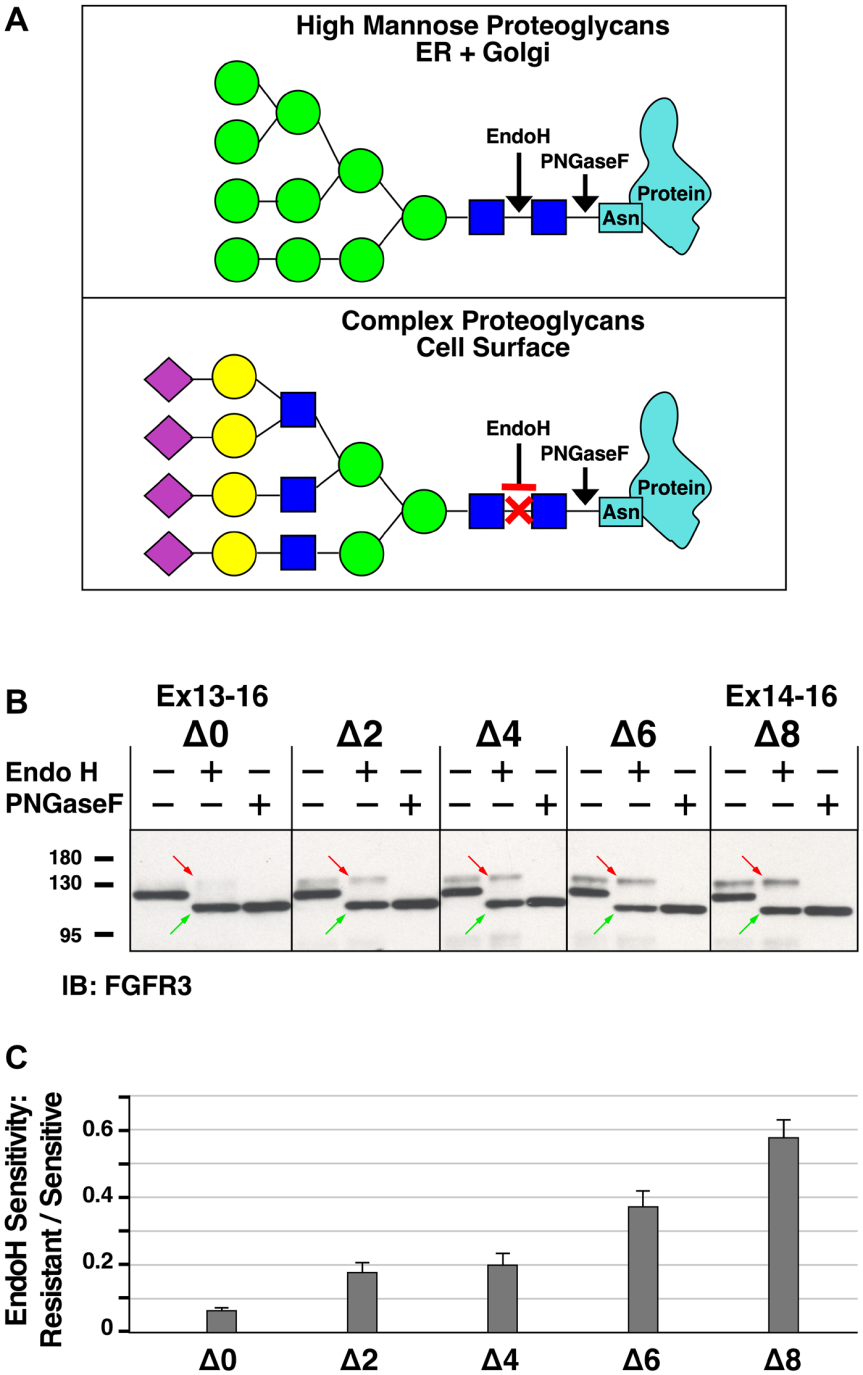
Analysis of Endoglycosidase H sensitivity of heptad deletion mutants of FGFR3-TACC3 Ex13-16. (**A**) Schematic showing cleavage sites of Endoglycosidase H (Endo H) and Peptide-N-Glycosylase F (PNGaseF) in typical proteoglycans. Monosaccharides are designated as in Glycopedia with blue squares designating GlcNAc, green circles representing mannose, yellow circles representing galactose, and purple diamonds representing sialic acid. (**B**) Endo H and PNGaseF digestions of heptad deletion mutants of FGFR3-TACC3 Ex13-16. Five representative samples were chosen from the complete set, and cell lysates from HEK293T cells were either untreated, digested with Endo H, or digested with PNGaseF followed by SDS-PAGE and immunoblotting. Upper bands indicated by the red arrows in the Endo H lane represent Endo H-resistant FGFR3-TACC3, whereas lower bands (green arrows) represents Endo H-sensitive FGFR3-TACC3. Note that the Endo H-sensitive FGFR3-TACC3 (green arrows) in the middle lane of each set comigrates with the complete digestion products generated by PNGaseF digestion, shown in the right lane of each set. (**C**) Quantitation of Endo H digestion products is presented as the ratio of Endo H-resistant versus Endo H-sensitive material in each of the samples treated with Endo H. Quantitation was obtained using immunoblots from four independent sets of digestions; ratios are presented as the mean +/− SEM. Molecular weight markers are indicated, kDa.

FGFR3 is a type I transmembrane protein that is cotranslationally inserted into the membrane in the endoplasmic reticulum (ER), and then transported through the Golgi to reach its final destination at the plasma membrane. The extracellular domain of FGFR3 undergoes cotranslational glycosylation to create a high mannose proteoglycan in the ER; this undergoes further sequential modification to form a complex proteoglycan on the cell surface where the glycans are terminated with sialic acid (see Figure 5A for simplified schematic). Elegant work from others has demonstrated that highly activated FGFR3 mutants are able to activate MAPK signaling from the endoplasmic reticulum [24, 25], thereby never reaching the cell surface before they are internalized and degraded, leading to an increased proportion of activated FGFR3 in a high mannose form compared to a complex proteoglycan.

We therefore analyzed the exon 13 heptad deletion mutants by comparing the high mannose forms of FGFR3-TACC3, whose glycans are sensitive to digestion by Endoglycosidase H (Endo H), with the population of FGFR3-TACC3 bearing complex proteoglycans, which are not sensitive to Endo H digestion (Figure 5B). A representative subset of the heptad mutants was chosen, including two transforming derivatives, Δ0 and Δ2, and three non-transforming mutants, Δ4, Δ6, and Δ8. The 1st lane of each set shows the native protein, the 2nd lane shows after digestion with Endo H, and the 3rd lane shows after digestion with PNGaseF, which removes all glycans including both high mannose and complex forms, leaving only the polypeptide core. Within the 2nd lane of each sample, a comparison of the upper band (EndoH-resistant, red arrow), with the lower band (EndoH-sensitive, green arrow), reveals the extent to which each population of FGFR3-TACC3 reaches the cell surface. Again, we see a general trend, with the transforming Δ0 mutant showing the lowest amount of EndoH-resistant protein, consistent with signaling and rapid removal from the ER [24, 25], and the non-transforming Δ8 mutant showing the greatest amount of EndoH-resistant protein, reflecting maturation of its proteoglycan during transit to the plasma membrane. The other mutants, Δ2, Δ4, and Δ6, fit this general trend, but again the correlation is somewhat anomalous, in that the transforming Δ2 mutant exhibits a very similar proportion of EndoH-resistant protein as does the non-transforming Δ4 mutant (Figure 5C).

## DISCUSSION

Through this work, we analyzed the downstream signaling, transforming abilities, and disulfide-bonded dimer formation of a selection of FGFR3-TACC3 fusion proteins with various breakpoints within TACC3 (Figure 1). Furthermore, we determined that FGFR3-TACC3 requires a minimum of five heptads from TACC3 exon 13 for biological activity (Figure 4).

For many of the experiments presented, we chose HEK293T cells as they allow for efficient protein expression from plasmid DNAs, a prerequisite for many experiments, and also for examination of downstream signaling pathways. Although originally thought to be of kidney epithelial cell origin, HEK293 cells and their HEK293T derivative [26] have been examined by gene expression profiling and are now thought to have originated from neural crest ectodermal cells in the embryonic adrenal medulla and thus have neuroectodermal properties [27, 28]. Given the frequent occurrence of FGFR3-TACC3 in glial cells, HEK293T cells may represent an appropriate cell line for the study of FGFR3-TACC3.

Although FGFR3-TACC3 Ex14-16 was previously detected in a head and neck cancer [17], this fusion protein was unable to demonstrate transforming ability (Figures 2A, 2B, 4B), nor form significant disulfide-bonded dimers (Figures 3A, 4D). Moreover, downstream signaling as reflected by phospho-MAPK showed a low level of activation in comparison with FGFR3-TACC3 Ex13-16 (Figures 2C, 4C). When compared to the biologically active fusion FGFR3-TACC3 Ex13-16, FGFR3-TACC3 Ex14-16 lacks eight heptads from its fusion partner TACC3, suggesting that these heptads are crucial for oncogenic activation. This provides a clear demonstration that not every presumptive fusion oncogene recovered from a human cancer will necessarily prove to be a driver of cellular proliferation.

In the biologically active FGFR3-TACC3 Ex13-16 fusion protein, a unique disulfide bond involving C828 was found to form intermolecularly within the C-terminal hydrophobic core of the coiled-coil dimerization interface (Figure 3A, 3D). Such positioning of a cysteine bond between coiled coils can significantly improve the stability of dimerization; this is often used as a tool for studying conformational changes arising from dimerization [29, 30], or for stabilization of rationally designed coiled-coil mimetics [31].

In addition to analyzing different FGFR3-TACC3 breakpoints for biological activity in transformation assays and for activation of MAPK signaling, we also analyzed sequential heptad deletions across exon 13 for the conversion of the high mannose form to a complex proteoglycan, a modification which occurs to the extracellular domain of FGFR3-TACC3. By comparing the EndoH-sensitive fraction with the EndoH-resistant fraction, we demonstrated that sequential heptad deletions across exon 13 led to a higher ratio of EndoH-resistant/sensitive protein (Figure 5C), which generally correlated with observed changes in biological activity and MAPK signaling assayed across the heptad series. This arises due to the ability of the transforming derivatives of FGFR3 to signal from the ER/Golgi which is accompanied by receptor internalization and degradation. Thus, a greater proportion of the non-transforming derivatives are expected to reach the plasma membrane as complex proteoglycans, which are resistant to digestion by Endo H but not by PNGaseF.

As disulfide-bonded coiled-coils are usually identified within the extracellular domain of proteins [32], it was unexpected to identify disulfide bonds within the cytoplasmic portion of FGFR3-TACC3, as the cytoplasm is generally considered a reducing environment that should favor thiol reduction. However, a novel metabolic function of FGFR3-TACC3 was recently identified as demonstrated by the ability of FGFR3-TACC3 to promote peroxisome biogenesis and new protein synthesis, which produce reactive oxygen species (ROS) [16]. Although redox-sensitive disulfide bonds have been identified that can regulate protein function in response to ROS levels [14, 22], we were unable to demonstrate a functional regulatory role for the disulfide bonds identified in FGFR3-TACC3 (data not shown).

One might wonder why the observed formation of disulfide bonded FGFR3-TACC3 correlates with the biological and signaling activity, even though mutation of the Cys residues involved in disulfide bond formation had no effect on biological activity (Figure 3E). We interpret the disulfide bond formation as a proxy for the stability of the FGFR3-TACC3 dimer; the more stable this dimer, which is formed through the interactions of the coiled-coil domains of TACC3 Ex13, the more likely that Cys residues 749 and 828 will undergo oxidation to form intermolecular disulfide bonds. However, we infer that the driving force for this dimerization remains the propensity of the coiled coils to interact.

Together, this work highlights the need to investigate different breakpoints of fusion proteins, as they may potentially lack the necessary biological activity required for oncogenesis, ultimately leading to misidentification of presumed oncogenes. Furthermore, many patients develop resistance to tyrosine kinase inhibitor (TKI) therapies, emphasizing the need for alternative therapeutic strategies which improve upon patient outcome. The rational design and use of a coiled-coil peptide-mimetic, which inhibits dimerization of TACC3 and subsequent activation of the FGFR3-TACC3 fusion protein, would be a beneficial therapeutic approach, as this would circumvent potential kinase-activating mutations that arise in the FGFR3 kinase domain. Taken together, these results provide a better understanding of the mechanism for activation of FGFR3-TACC3 and narrow the scope of targeting TACC3 to create effective dimerization disruption-based therapies for treating patients with FGFR3-TACC3 driven tumors.

## MATERIALS AND METHODS

### DNA constructs

The FGFR3-TACC3 gene was constructed as described previously [3, 8]. Single and double mutations of cysteines in FGFR3-TACC3 were introduced by PCR-based site-directed mutagenesis. Heptads were deleted from FGFR3-TACC3 by PCR site-directed mutagenesis, then restriction enzyme digests, and finally internal ligations. Each construct was subcloned to create two versions with pcDNA3 or pLXSN vectors [33].

### Cell culture, transfection, and immunoblotting

NIH3T3 cells (RRID:CVCL_0594) were maintained in 10% Calf Serum (CS) in DMEM media with 1% penicillin/streptomycin in 10% CO_2_ at 37°C [8]. NIH3T3 cells were obtained in approximately 1982 from the laboratory of Prof. Robert A. Weinberg of Massachusetts Institute of Technology. NIH3T3 cells were authenticated by murine STR profiling conducted by ATCC, using FTA Sample Collection Kit for Mouse Cell Authentication Service (ATCC #137-XV), and found to be a 100% match with ATCC control cell line CRL-1658 (FTA Barcode MUSA1230).

HEK293T cells (RRID:CVCL_0063) were maintained in 10% Fetal Bovine Serum (FBS) in DMEM media with 1% penicillin/streptomycin in 10% CO_2_ at 37°C. HEK293T cells were obtained in 1998 from the laboratory of Prof. Thomas J. Hope, then at the Salk Institute for Biological Studies, currently at Northwestern University Feinberg School of Medicine. HEK293T cells were authenticated by human short tandem repeat (STR) profiling conducted by American Type Culture Collection (ATCC), using FTA Sample Collection Kit for Human Cell Authentication (ATCC #135-XV). Results indicated a 93% match with ATCC control cell line CRL-3216 (FTA Barcode STRC3156), a variation which may be due to microsatellite instability. All cells were verified as Mycoplasma negative using the MycoStrip Mycoplasma detection kit (InvivoGen).

Twenty-four hours before transfection HEK293T cells were plated at a density of 1 × 10^6^ cells per 100 mm plate. These cells were transfected with pcDNA3 constructs by calcium phosphate transfection as described previously [3]. Cells were then incubated in 3% CO_2_ at 37°C for 17 hours and recovered via incubation in 10% CO_2_ at 37°C for 6–8 hours. These cells were serum deprived in 0% FBS/DMEM for 16 hours. Afterwards, cells were washed in 1× ice-cold PBS and then lysed in radioimmunoprecipitation assay buffer [RIPA; 50 mM Tris-HCl (pH 8.0), 150 mM NaCl, 1% TritionX-100, 0.5% sodium deoxycholate, 0.1% SDS, 50 mM NaF, 1 mM sodium orthovanadate, 0.1 mM PMSF, and 10 μg/mL aprotinin]. Lowry assay was used to measure total protein concentration. Samples were separated by 12.5% SDS-PAGE and transferred to Immobilon-P membranes (Millipore, Burlington, MA, USA). Membranes were blocked in 3% milk/TBS/0.05% Tween 20 or 3–5% bovine serum albumin (BSA)/TBS/0.05% Tween 20. Antibodies were added to lysates for overnight incubation at 4°C with rocking [8].

For dimerization assays, cells were washed twice with 1× PBS containing 10 mM iodoacetamide and then lysed in RIPA-Plus Buffer (10 mM sodium phosphate pH 7.0, 150 mM sodium chloride, 1% Triton X-100, 0.1% SDS, 1% sodium deoxycholate, 2 mM EDTA, 50 mM NaF, 10 mM iodoacetamide, 10 μg/ml aprotinin, 1 mM sodium orthovanadate, 0.1 mM PMSF). Lysates were boiled in 2× non-reducing sample buffer (4% SDS, 10 mM sodium phosphate pH 7.0, 20% glycerol, 0.08% bromophenol blue) after which half of the sample was transferred to a new tube and reduced with 5% β-mercaptoethanol and 20 mM DTT [23]. Samples were resolved on 4–12% SDS-PAGE gradient gels and then transferred to Immobilon-P membrane. Membranes were blocked in 5% milk/TBS/0.05% Tween 20 and immunoblotted as described above. For experiments that examine the effects of intracellular ROS on FGFR3-TACC3 dimer formation and receptor activation, cells were treated with either H_2_O_2_ or Diamide as described [15]. For glycosylation analysis, lysates from HEK293T transfected cells were treated according to manufacturer directions and separated by 12.5% SDS-PAGE and immunoblotted as above.

The following antibodies and reagents were used for immunoblotting: N-terminal FGFR3 (B-9) (Santa Cruz Biotechnology, Dallas, TX, USA); C-terminal TACC3 (SAB4500103) (Sigma-Aldrich, St. Louis, MO, USA); Phospho-FGF Receptor (Y653/654) (#3471); Phospho-p44/42 MAPK (T202/Y204) (D13.14.4E) (#4370); p44/42 MAPK (Erk1/2) (#9102) (Cell Signaling Technology, Danvers, MA, USA); horseradish peroxidase (HRP) anti-mouse; HRP anti-rabbit and Enhanced Chemiluminescence (ECL) reagents (GE Healthcare, Little Chalfont, UK). Endoglycosidase H (Endo-H) and Peptide-N-Glycosidase F (PNGase F) were obtained from New England Biolabs (Ipswich, MA, USA).

NIH3T3 cells were plated to a density of 4 × 10^5^ cells per 60 mm plate 1 day prior to transfection with pLXSN constructs as described [8]. These cells were then transfected with 10 μg of pLXSN constructs using Lipofectamine 2000 reagent (Invitrogen, Carlsbad, CA, USA). Sixteen hours following transfection, cells were refed with 10% CS/DMEM. Forty-eight hours following transfection, cells were split 1:12 onto duplicate 100 mm plates containing 2.5% CS/DMEM. Efficiency of transfection was determined by Geneticin (G418, 0.5 mg/mL)-resistant colonies plated on duplicate plates at a dilution of 1:240 in 10% CS/DMEM. Eighteen days following transfection, plates were fixed with methanol, stained with Giemsa stain, and scored. The foci were normalized against the G418 colony counts for transfection efficiency [8].

## References

[1] Gallo LH , Nelson KN , Meyer AN , Donoghue DJ . Functions of Fibroblast Growth Factor Receptors in cancer defined by novel translocations and mutations. Cytokine Growth Factor Rev. 2015; 26:425–49. 10.1016/j.cytogfr.2015.03.003. 26003532

[2] Peiris MN , Li F , Donoghue DJ . BCR: a promiscuous fusion partner in hematopoietic disorders. Oncotarget. 2019; 10:2738–54. 10.18632/oncotarget.26837. 31105873 PMC6505627

[3] Nelson KN , Meyer AN , Siari A , Campos AR , Motamedchaboki K , Donoghue DJ . Oncogenic Gene Fusion FGFR3-TACC3 Is Regulated by Tyrosine Phosphorylation. Mol Cancer Res. 2016; 14:458–69. 10.1158/1541-7786.MCR-15-0497. 26869289

[4] Li F , Meyer AN , Peiris MN , Nelson KN , Donoghue DJ . Oncogenic fusion protein FGFR2-PPHLN1: Requirements for biological activation, and efficacy of inhibitors. Transl Oncol. 2020; 13:100853. 10.1016/j.tranon.2020.100853. 32854034 PMC7451725

[5] Singh D , Chan JM , Zoppoli P , Niola F , Sullivan R , Castano A , Liu EM , Reichel J , Porrati P , Pellegatta S , Qiu K , Gao Z , Ceccarelli M , et al. Transforming fusions of FGFR and TACC genes in human glioblastoma. Science. 2012; 337:1231–35. 10.1126/science.1220834. 22837387 PMC3677224

[6] Parker BC , Annala MJ , Cogdell DE , Granberg KJ , Sun Y , Ji P , Li X , Gumin J , Zheng H , Hu L , Yli-Harja O , Haapasalo H , Visakorpi T , et al. The tumorigenic FGFR3-TACC3 gene fusion escapes miR-99a regulation in glioblastoma. J Clin Invest. 2013; 123:855–65. 10.1172/JCI67144. 23298836 PMC3561838

[7] Sarkar S , Ryan EL , Royle SJ . FGFR3-TACC3 cancer gene fusions cause mitotic defects by removal of endogenous TACC3 from the mitotic spindle. Open Biol. 2017; 7:170080. 10.1098/rsob.170080. 28855393 PMC5577446

[8] Nelson KN , Meyer AN , Wang CG , Donoghue DJ . Oncogenic driver FGFR3-TACC3 is dependent on membrane trafficking and ERK signaling. Oncotarget. 2018; 9:34306–19. 10.18632/oncotarget.26142. 30344944 PMC6188140

[9] Nelson KN , Peiris MN , Meyer AN , Siari A , Donoghue DJ . Receptor Tyrosine Kinases: Translocation Partners in Hematopoietic Disorders. Trends Mol Med. 2017; 23:59–79. 10.1016/j.molmed.2016.11.002. 27988109

[10] Chell V , Balmanno K , Little AS , Wilson M , Andrews S , Blockley L , Hampson M , Gavine PR , Cook SJ . Tumour cell responses to new fibroblast growth factor receptor tyrosine kinase inhibitors and identification of a gatekeeper mutation in FGFR3 as a mechanism of acquired resistance. Oncogene. 2013; 32:3059–70. 10.1038/onc.2012.319. 22869148

[11] Peiris MN , Meyer AN , Nelson KN , Bisom-Rapp EW , Donoghue DJ . Oncogenic fusion protein BCR-FGFR1 requires the breakpoint cluster region-mediated oligomerization and chaperonin Hsp90 for activation. Haematologica. 2020; 105:1262–73. 10.3324/haematol.2019.220871. 31439673 PMC7193502

[12] Dixon AS , Pendley SS , Bruno BJ , Woessner DW , Shimpi AA , Cheatham TE 3rd , Lim CS . Disruption of Bcr-Abl coiled coil oligomerization by design. J Biol Chem. 2011; 286:27751–60. 10.1074/jbc.M111.264903. 21659527 PMC3149365

[13] Woessner DW , Eiring AM , Bruno BJ , Zabriskie MS , Reynolds KR , Miller GD , O’Hare T , Deininger MW , Lim CS . A coiled-coil mimetic intercepts BCR-ABL1 dimerization in native and kinase-mutant chronic myeloid leukemia. Leukemia. 2015; 29:1668–75. 10.1038/leu.2015.53. 25721898 PMC4621806

[14] Zuo Y , Xiang B , Yang J , Sun X , Wang Y , Cang H , Yi J . Oxidative modification of caspase-9 facilitates its activation via disulfide-mediated interaction with Apaf-1. Cell Res. 2009; 19:449–57. 10.1038/cr.2009.19. 19238172

[15] Cumming RC , Andon NL , Haynes PA , Park M , Fischer WH , Schubert D . Protein disulfide bond formation in the cytoplasm during oxidative stress. J Biol Chem. 2004; 279:21749–58. 10.1074/jbc.M312267200. 15031298

[16] Frattini V , Pagnotta SM , Tala , Fan JJ , Russo MV , Lee SB , Garofano L , Zhang J , Shi P , Lewis G , Sanson H , Frederick V , Castano AM , et al. A metabolic function of FGFR3-TACC3 gene fusions in cancer. Nature. 2018; 553:222–27. 10.1038/nature25171. 29323298 PMC5771419

[17] Yuan L , Liu ZH , Lin ZR , Xu LH , Zhong Q , Zeng MS . Recurrent FGFR3-TACC3 fusion gene in nasopharyngeal carcinoma. Cancer Biol Ther. 2014; 15:1613–21. 10.4161/15384047.2014.961874. 25535896 PMC4622012

[18] Williams SV , Hurst CD , Knowles MA . Oncogenic FGFR3 gene fusions in bladder cancer. Hum Mol Genet. 2013; 22:795–803. 10.1093/hmg/dds486. 23175443 PMC3554204

[19] Bielle F , Di Stefano AL , Meyronet D , Picca A , Villa C , Bernier M , Schmitt Y , Giry M , Rousseau A , Figarella-Branger D , Maurage CA , Uro-Coste E , Lasorella A , et al. Diffuse gliomas with FGFR3-TACC3 fusion have characteristic histopathological and molecular features. Brain Pathol. 2018; 28:674–83. 10.1111/bpa.12563. 28976058 PMC8028622

[20] Trigg J , Gutwin K , Keating AE , Berger B . Multicoil2: predicting coiled coils and their oligomerization states from sequence in the twilight zone. PLoS One. 2011; 6:e23519. 10.1371/journal.pone.0023519. 21901122 PMC3162000

[21] Thiyagarajan N , Bunney TD , Katan M . 5LXN Coiled-coil protein. PDB. 2016. 10.2210/pdb5LXN/pdb.

[22] Wouters MA , Fan SW , Haworth NL . Disulfides as redox switches: from molecular mechanisms to functional significance. Antioxid Redox Signal. 2010; 12:53–91. 10.1089/ars.2009.2510. 19634988

[23] Chen LI , Webster MK , Meyer AN , Donoghue DJ . Transmembrane domain sequence requirements for activation of the p185c-neu receptor tyrosine kinase. J Cell Biol. 1997; 137:619–31. 10.1083/jcb.137.3.619. 9151669 PMC2139875

[24] Lievens PM , Mutinelli C , Baynes D , Liboi E . The kinase activity of fibroblast growth factor receptor 3 with activation loop mutations affects receptor trafficking and signaling. J Biol Chem. 2004; 279:43254–60. 10.1074/jbc.M405247200. 15292251

[25] Lievens PM , Roncador A , Liboi E . K644E/M FGFR3 mutants activate Erk1/2 from the endoplasmic reticulum through FRS2 alpha and PLC gamma-independent pathways. J Mol Biol. 2006; 357:783–92. 10.1016/j.jmb.2006.01.058. 16476447

[26] DuBridge RB , Tang P , Hsia HC , Leong PM , Miller JH , Calos MP . Analysis of mutation in human cells by using an Epstein-Barr virus shuttle system. Mol Cell Biol. 1987; 7:379–87. 10.1128/mcb.7.1.379-387.1987. 3031469 PMC365079

[27] Lin YC , Boone M , Meuris L , Lemmens I , Van Roy N , Soete A , Reumers J , Moisse M , Plaisance S , Drmanac R , Chen J , Speleman F , Lambrechts D , et al. Genome dynamics of the human embryonic kidney 293 lineage in response to cell biology manipulations. Nat Commun. 2014; 5:4767. 10.1038/ncomms5767. 25182477 PMC4166678

[28] Stepanenko AA , Dmitrenko VV . HEK293 in cell biology and cancer research: phenotype, karyotype, tumorigenicity, and stress-induced genome-phenotype evolution. Gene. 2015; 569:182–90. 10.1016/j.gene.2015.05.065. 26026906

[29] Anthony LC , Dombkowski AA , Burgess RR . Using disulfide bond engineering to study conformational changes in the beta’260-309 coiled-coil region of Escherichia coli RNA polymerase during sigma(70) binding. J Bacteriol. 2002; 184:2634–41. 10.1128/JB.184.10.2634-2641.2002. 11976292 PMC135008

[30] Weitzel CS , Waldman VM , Graham TA , Oakley MG . A repeated coiled-coil interruption in the Escherichia coli condensin MukB. J Mol Biol. 2011; 414:578–95. 10.1016/j.jmb.2011.10.028. 22041452

[31] Wuo MG , Mahon AB , Arora PS . An Effective Strategy for Stabilizing Minimal Coiled Coil Mimetics. J Am Chem Soc. 2015; 137:11618–21. 10.1021/jacs.5b05525. 26340721 PMC4577959

[32] Thornton JM . Disulphide bridges in globular proteins. J Mol Biol. 1981; 151:261–87. 10.1016/0022-2836(81)90515-5. 7338898

[33] Miller AD , Rosman GJ . Improved retroviral vectors for gene transfer and expression. Biotechniques. 1989; 7:980–82. 2631796 PMC1360503

